# Low SARS-CoV-2 Transmission in Elementary Schools — Salt Lake County, Utah, December 3, 2020–January 31, 2021

**DOI:** 10.15585/mmwr.mm7012e3

**Published:** 2021-03-26

**Authors:** Rebecca B. Hershow, Karen Wu, Nathaniel M. Lewis, Alison T. Milne, Dustin Currie, Amanda R. Smith, Spencer Lloyd, Brian Orleans, Erin L. Young, Brandi Freeman, Noah Schwartz, Bobbi Bryant, Catherine Espinosa, Yoshinori Nakazawa, Elizabeth Garza, Olivia Almendares, Winston E. Abara, Daniel C. Ehlman, Keith Waters, Mary Hill, Ilene Risk, Kelly Oakeson, Jacqueline E. Tate, Hannah L. Kirking, Angela Dunn, Snigdha Vallabhaneni, Adam L. Hersh, Victoria T. Chu

**Affiliations:** ^1^CDC COVID-19 Response Team; ^2^Epidemic Intelligence Service, CDC; ^3^Utah Department of Health; ^4^Granite School District, Salt Lake City, Utah; ^5^Health and Economic Recovery Outreach (HERO) Project, University of Utah Health Sciences, Salt Lake City, Utah; ^6^Utah Public Health Laboratory, Taylorsville, Utah; ^7^General Dynamics Information Technology, Falls Church, Virginia; ^8^Salt Lake County Health Department, Salt Lake City, Utah.

School closures affected more than 55 million students across the United States when implemented as a strategy to prevent the transmission of SARS-CoV-2, the virus that causes COVID-19 (*1*). Reopening schools requires balancing the risks for SARS-CoV-2 infection to students and staff members against the benefits of in-person learning (*2*). During December 3, 2020–January 31, 2021, CDC investigated SARS-CoV-2 transmission in 20 elementary schools (kindergarten through grade 6) that had reopened in Salt Lake County, Utah. The 7-day cumulative number of new COVID-19 cases in Salt Lake County during this time ranged from 290 to 670 cases per 100,000 persons.^†^ Susceptible^§^ school contacts^¶^ (students and staff members exposed to SARS-CoV-2 in school) of 51 index patients** (40 students and 11 staff members) were offered SARS-CoV-2 reverse transcription–polymerase chain reaction (RT-PCR) testing. Among 1,041 susceptible school contacts, 735 (70.6%) were tested, and five of 12 cases identified were classified as school-associated; the secondary attack rate among tested susceptible school contacts was 0.7%. Mask use among students was high (86%), and the median distance between students’ seats in classrooms was 3 ft. Despite high community incidence and an inability to maintain ≥6 ft of distance between students at all times, SARS-CoV-2 transmission was low in these elementary schools. The results from this investigation add to the increasing evidence that in-person learning can be achieved with minimal SARS-CoV-2 transmission risk when multiple measures to prevent transmission are implemented (*3*,*4*).

On August 24, 2020, a school district in Salt Lake County, Utah, reopened schools for in-person learning.^††^Elementary schools restricted school-related extracurricular activities and large group gatherings, placed students in cohorts by classroom, and implemented other COVID-19 strategies to limit spread.^§§^ During December 3, 2020–January 31, 2021, CDC was invited by the Utah Department of Health to investigate SARS-CoV-2 transmission in a convenience sample of 20 elementary schools in partnership with the school district, the University of Utah’s Health and Economic Recovery Outreach (HERO) Project,^¶¶^ Utah Department of Health, and Salt Lake County Health Department.

School contacts of identified index patients completed a questionnaire about symptoms and exposures and received SARS-CoV-2 testing. Written consent was provided by participants (or by a parent or guardian for minors). Persons not susceptible to SARS-CoV-2 infection were excluded. Saliva samples (or nasal swabs if saliva was unobtainable) were collected for SARS-CoV-2 RT-PCR testing 5–10 days postexposure; turnaround time for results was typically 1–2 days. Household members of school contacts with a positive SARS-CoV-2 test result were interviewed and offered SARS-CoV-2 RT-PCR testing. The Utah Public Health Laboratory performed whole genome sequencing (WGS) for available positive specimens. A school contact who received a positive test result was considered not to have a school-associated case of COVID-19 when one of the following occurred: 1) illness onset preceded the first date of school exposure, 2) a household member had illness onset during the 14 days preceding the school contact’s illness onset (for symptomatic school contacts) or before the last date of school exposure (for asymptomatic school contacts), or 3) WGS demonstrated that the lineage of the index patient’s isolate differed from that of the school contact.*** To understand school mitigation measures and classroom characteristics, principals and teachers of each index patient were surveyed. Classroom seat distances between students and between the teacher and nearest student were measured. SAS (version 9.4; SAS Institute) was used for descriptive statistics. This activity was reviewed by CDC and was conducted consistent with applicable federal law and CDC policy.^†††^

The 20 elementary schools included 1,214 staff members and 10,171 students, 81% of whom attended school in person and 56% of whom were eligible for free or reduced-price meal programs. Among the student population, 53% were non-Hispanic White persons, 31% were Hispanic or Latino persons, 5% were Asian persons, 5% were Native Hawaiian or Other Pacific Islander persons, and 4% were Black or African American persons. Fifty-one index patients (40 students, median age = 9.5 years [range = 5–12 years] and 11 staff members, median age = 50 years [range = 26–62 years]) were identified from 48 classrooms (Table 1). These index patients were infectious at school for a median of 2 days (range = 1–4 days), and 16 (31%) were asymptomatic. A total of 1,083 school contacts (943 students and 140 staff members) were identified; 42 (4%) were not susceptible to SARS-CoV-2 infection.^§§§^ Among the 1,041 susceptible school contacts (student median age = 9 years [range = 5–18 years]; staff member median age = 39.5 years [range = 19–83 years]), 144 (14%) were quarantined (Table 2). Among the 735 (71%) tested school contacts (participation range = 44%–100% across schools), testing was completed a median of 8 days after the school exposure (range = 6–15 days). Overall, 103 of 133 (77%) staff member contacts and 632 of 908 (70%) student contacts were tested; among 303 Hispanic or Latino contacts and 566 non-Hispanic White contacts, 237 (78%) and 382 (67%) respectively, were tested.

**TABLE 1:** Characteristics of index and school-associated patients with laboratory-confirmed COVID-19 in 20 elementary schools — Salt Lake County, Utah, December 3, 2020–January 31, 2021

Characteristic	No. (%) of persons with COVID-19	
	Index (n = 51)*	School-associated (n = 5)^†^
**Cases per school, median (range)**	2 (1–9)	0 (0–2)
**School contacts, median (range)**	20 (5–53)	—**^§^**
Close contacts, median (range)	6 (0–23)	—
Other school contacts, median (range)	13 (0–52)	—
**Median age, yrs (range)**		
Students (index: n = 40; school-associated: n = 4)	9.5 (5–12)	10.5 (10–12)
Staff members (index: n = 11; school-associated: n = 1)	50 (26–62)	43 (43–43)
**Sex**		
Male	24 (47.1)	2 (40.0)
Female	27 (52.9)	3 (60.0)
**Race/Ethnicity**		
White, non-Hispanic	30 (58.8)	1 (20.0)
Hispanic/Latino	15 (29.4)	2 (40.0)
Black/African American	1 (2.0)	0 (0.0)
Asian	1 (2.0)	1 (20.0)
Native Hawaiian/Other Pacific Islander	2 (3.9)	0 (0.0)
American Indian or Alaska Native	0 (0.0)	0 (0.0)
Multiracial	2 (3.9)	1 (20.0)
**Grade in school^¶^**		
Kindergarten	5 (12.5)	0 (0.0)
1	3 (7.5)	0 (0.0)
2	2 (5.0)	0 (0.0)
3	6 (15.0)	0 (0.0)
4	6 (15.0)	2 (50.0)
5	8 (20.0)	0 (0.0)
6	10 (25.0)	2 (50.0)
**Role in school**		
Students	40 (78.4)	4 (80.0)
Head teachers	6 (11.8)	1 (20.0)
Paraeducators**	0 (0.0)	0 (0.0)
Other teachers^††^	4 (7.8)	0 (0.0)
Other staff members^§^**^§^**	1 (2.0)	0 (0.0)
**Days in school while infectious, median (range)**	2 (1–4)	0 (0–2)
**Symptom status**		
Ever symptomatic	35 (68.6)	2 (40.0)
Asymptomatic	16 (31.4)	3 (60.0)
One or more underlying medical condition^¶¶^	9 (20.9)	0 (0.0)
Quarantine status after exposure to index patient***		
Under quarantine	—	3 (60.0)
Notified, close contact	—	0 (0.0)
Notified, not close contact	—	2 (40.0)

**Abbreviation:** IQR= interquartile range.

* An index patient was defined as a student or staff member with laboratory-confirmed SARS-CoV-2 infection who had attended in-person school while infectious for at least 1 day. Infectious period was estimated as 2 days before to 10 days after symptom onset (if symptomatic) or first positive specimen collection date (if asymptomatic).

^†^ School-associated transmission was excluded if 1) the school contact had an illness onset (if symptomatic, symptom onset, if asymptomatic, first positive test date) before the last date of school exposure, 2) a household member had an illness onset (if symptomatic, symptom onset, if asymptomatic, first positive test date) within 14 days of the positive school contact’s illness onset (if school contact was symptomatic) or before the last date of school exposure (if the school contact was asymptomatic) or 3) whole genome sequencing supported nonschool-associated transmission.

^§^ Dashes indicate that data are not applicable.

^¶^ Restricted to students. For index patients, n = 40, for secondary cases, n = 4.

** Includes teacher aides and interns.

^††^ Includes ethics teachers, instructional coaches, learning support teachers, special education teachers, and substitute teachers.

^§§^ Includes administrators, bus drivers, and health specialists.

^¶¶^ Missing data: Underlying medical conditions: eight index patients, one school-associated patient.

*** Starting January 4, 2021, the school district changed its quarantine policy based on changes to state recommendations, and only students and staff members identified as close contacts (i.e., within 6 ft of the index patient for a cumulative total of ≥15 minutes over a 24-hour period) of the index patient were quarantined when both were maskless; previously, all close contacts would have been quarantined regardless of mask use. Any close contacts identified in January who met the criteria to not quarantine were categorized as “Notified, close contact.” Those who shared a classroom space with the index patient but were not identified as close contacts were categorized as “Notified, not close contact.”

**TABLE 2:** Characteristics of COVID-19–susceptible school contacts* in 20 elementary schools — Salt Lake County, Utah, December 3, 2020–January 31, 2021

Characteristic	No. (%) of school contacts	
	Total (N = 1,041)	Tested (n = 735)
**Overall participation**	—**^†^**	735 (70.6)
**Median percent participation across 20 schools (range)**	—	69.7 (44.4–100.0)
**Median age, yrs (range)^§^**		
Students (n = 908)	9.0 (5.0–18.0)	9.0 (5.0–18.0)
Staff members (n = 112)	39.5 (19.0–83.0)	39.0 (19.0–83.0)
**Sex**		
Male	487 (47.7)	352 (47.9)
Female	535 (52.3)	383 (52.1)
**Race/Ethnicity**		
White, non-Hispanic	566 (55.9)	382 (52.0)
Hispanic/Latino	303 (29.9)	237 (32.2)
Black/African American	28 (2.8)	25 (3.4)
Asian	33 (3.3)	29 (3.9)
Native Hawaiian/Other Pacific Islander	28 (2.8)	15 (2.0)
American Indian or Alaska Native	8 (0.8)	7 (1.0)
Multiracial	47 (4.6)	40 (5.4)
**Grade** ^¶^		
Kindergarten	110 (12.1)	61 (9.7)
1	107 (11.8)	79 (12.5)
2	139 (15.3)	108 (17.1)
3	113 (12.4)	78 (12.3)
4	134 (14.8)	95 (15.0)
5	118 (13.0)	86 (13.6)
6	182 (20.0)	121 (19.1)
≥7	5 (0.6)	4 (0.6)
**Role in school**		
Students	908 (87.2)	632 (86.0)
Head teachers	77 (7.4)	61 (8.3)
Paraeducators**	24 (2.3)	13 (1.8)
Other teachers**^††^**	14 (1.3)	12 (1.6)
Other staff members^§§^	18 (1.7)	17 (2.3)
**Days between school exposure and test date, median (range)** ^¶¶^	8 (6–15)	8 (6–15)
Quarantine status after exposure to index patient***		
Quarantined	144 (13.8)	105 (14.3)
Notified, close contact	183 (17.6)	131 (17.8)
Notified, not close contact	714 (68.6)	499 (67.9)

* School contact was defined as a student or staff member who was in contact with the index patient for a total of ≥15 minutes in a classroom, cafeteria, school bus, or recess space during an index patient’s infectious period. This includes any contacts who received positive SARS-CoV-2 test results but were not determined to have school-associated cases.

^†^ Dashes indicate that data are not applicable.

^§^ Missing data (also applies to Sex and Race/Ethnicity categories): Age: 21 nonparticipating staff members; Sex: 19 nonparticipating staff members; Race/Ethnicity: 28 nonparticipants.

^¶^ Restricted to students (n = 908). Students in grade 7 or higher were contacts of an elementary school student on the school bus. All five students in grade 7 or higher were contacts of the same index patient. Bus contacts were not routinely included on the list of school contacts for all 51 index patients.

** Includes teacher aides and interns.

^††^ Includes ethics teachers, instructional coaches, learning support teachers, special education teachers, and substitute teachers.

^§§^ Includes administrators, bus drivers, and health specialists.

^¶¶^ All classroom testing occurred 6–10 days after exposure. One contact was tested on day 8 and offered a follow-up repeat testing on day 15.

*** Starting January 4, 2021, the school district changed its quarantine policy based on changes to state recommendations, and only students and staff members identified as close contacts (i.e., within 6 ft of the index patient for a cumulative total of ≥15 minutes over a 24-hour period) of the index patient were quarantined when both were maskless; previously, all close contacts would have been quarantined regardless of mask use. Any close contacts identified in January who met the criteria to not quarantine were categorized as “Notified, close contact.” Those who shared a classroom space with the index patient but were not identified as close contacts were categorized as “Notified, not close contact.”

Among all 735 tested contacts, 12 (1.6%) (11 students, one teacher) had a positive SARS-CoV-2 test result, seven of whom were determined not to have school-associated cases because of epidemiologic evidence (four) or because WGS suggested community acquisition based on lineage differences (three) (Supplementary Figure, https://stacks.cdc.gov/view/cdc/104112). WGS was only available for three pairs of index patients and their associated contacts (Table 3). After exclusion, five cases from five separate classrooms were classified as school-associated, for a secondary attack rate of 0.7% (five of 728). No outbreaks were detected.^¶¶¶^Three of five persons with school-associated cases had been quarantined (the secondary attack rate among quarantined persons who were tested was 3.0% [three of 101]); the remaining two persons with school-associated cases had not been quarantined and were isolated only after a positive test result (secondary attack rate among nonquarantined contacts who were tested = 0.3% [two of 627]).**** Among the five persons with school-associated cases, three persons were asymptomatic, and three persons were exposed to asymptomatic index patients; four cases were attributed to student-to-student transmission, and one was attributed to student-to-teacher transmission. Four of the five school-associated transmission events occurred because the contact sat <6 ft from the index patient during class (two) or during lunch (two), or the index patient or contact had poor mask use (two) or physical distancing behavior (two) (Table 3). All five households of persons with school-associated cases were tested. Tertiary transmission was detected in three households; within those households, six of eight household members received positive SARS-CoV-2 test results.

**TABLE 3:** Characteristics of 12 contacts who received positive SARS-CoV-2 test results and summary of evidence for school-associated transmission in five contacts across 20 elementary schools — Salt Lake County, Utah, December 3, 2020–January 31, 2021*

Positive contact ID	Index patient		School contact^†^		School-associated transmission			Factors associated with transmission			
	School role	Symptoms reported	School role	Symptoms reported	Basis for exclusion of school-associated transmission		School-associated transmission hypothesized	Close contact between patient and contact^†^	Contact sat <6 ft from index patient	Poor adherence to distancing, mask use, or neither at school	
					Epidemiologic data	WGS data				Index patient	Contact
I1	Student	N	Student	N	N	NA	Y	Y	Class	Distancing	Mask use, distancing
J2	Student	N	Student	Y	N	NA	Y	Y	Class	Neither	Mask use
X3	Student	Y	Student	N	N	NA	Y	N	Lunch	Neither	Distancing
AA4	Student	Y	Student	N	N	NA	Y	Y	Lunch	Neither	Neither
EE5	Student	N	Teacher	Y	N	NA	Y	N	Neither	Neither	Neither
A6	Student	Y	Student	Y	N	Y	N	Y	—^§^	—	—
A7	Student	Y	Student	N	N	Y	N	Y	—	—	—
L8	Student	N	Student	Y	N	Y	N	Y	—	—	—
O9	Teacher	N	Student	Y	Y	NA	N	Y	—	—	—
T10	Student	Y	Student	Y	Y	NA	N	Y	—	—	—
RR11	Teacher	Y	Student	Y	Y	NA	N	Y	—	—	—
VV12	Student	Y	Student	Y	Y	NA	N	Y	—	—	—

**Abbreviations:** ID = identifier; Y = yes; N = no; NA = not available; WGS = whole genome sequencing.

* School-associated transmission was excluded by epidemiologic data if 1) the school contact had an illness onset (if symptomatic, symptom onset; if asymptomatic, first positive test date) before the last date of school exposure, or 2) a household member had an illness onset (if symptomatic, symptom onset; if asymptomatic, first positive test date) within 14 days of the positive school contact’s illness onset (if school contact was symptomatic) or before the last date of school exposure (if the school contact was asymptomatic). School-associated transmission was excluded by WGS data if the index patient isolate was found to be a different lineage from the positive school contact isolate.

^†^ Persons were determined to be close contacts if they were <6 ft from the index patient for a cumulative total of ≥15 minutes during a 24-hour period at school. All other school contacts were students or staff members who were in contact with the index patient for a cumulative total of ≥15 minutes in a classroom, cafeteria, school bus, or recess space during an index patient’s infectious period.

^§^ Dashes indicate that data are not applicable.

On December 17, 2020, Utah modified its quarantine recommendations for school contacts (students or staff members) who were identified as close contacts (persons within 6 ft of the index patient for a cumulative total of ≥15 minutes during a 24-hour period). Previously, school contacts who were close contacts were quarantined^††††^ regardless of mask use; afterwards, they were only quarantined when the index patient or the contact did not wear a mask during the interaction. The school district implemented this recommendation on January 4, 2021, after a holiday break, and 158 students who were close contacts continued attending in-person school. Among these 158 students, 111 (70%) were tested; no school-associated cases were detected.

Students in 42 classrooms^§§§§^ (median class size = 22 students [range = 3–33 students]) sat a median of 3 ft (range = 1–5 ft) apart within the classroom, with a median of eight students (range = 1–16 students) sitting within a radius of 6 ft (Supplementary Table 1, https://stacks.cdc.gov/view/cdc/104112). Among 37 teachers with available data, 23 (62%) were seated ≥6 ft from the closest student (median = 6 ft, range = 2–10 ft), but all teachers reported daily one-on-one or small group instruction in close proximity to students, almost always without using plexiglass or physical barriers. Among 42 teachers, 36 (86%) reported that students always wore masks indoors except when eating or drinking. Nineteen of 20 (95%) principals reported using staggered mealtimes to increase spacing between students during lunch in the cafeteria (although still <6 ft apart). All schools reported implementing multiple measures to decrease in-school SARS-CoV-2 transmission (Supplementary Table 2, https://stacks.cdc.gov/view/cdc/104112).

## Discussion

Despite high community incidence and an inability to space students’ classroom seats ≥6 ft apart, this investigation found low SARS-CoV-2 transmission and no school-related outbreaks in 20 Salt Lake County elementary schools with high student mask use and implementation of multiple strategies to limit transmission. Other U.S. studies have also detected minimal school-associated transmission when implementing strict mitigation measures, although testing was limited to symptomatic close contacts (*3*,*4*). Because children with COVID-19 are frequently asymptomatic (*5*), the expanded testing to all school contacts regardless of symptom status in this investigation strengthens the evidence for low elementary school transmission.

In addition to implementation of multiple strategies to reduce in-school transmission, school-related activities that increase the risk for SARS-CoV-2 transmission, such as school-based team sports (*6*), were suspended. Although most teachers were seated ≥6 ft from students, CDC’s recommendation at the time of the study of ≥6 ft student distancing within the classroom (*7*) was not possible because of limited space. A recent study in Massachusetts found no difference in student and staff member case rates from school districts with ≥3 feet physical distancing requirements compared with school districts with ≥6 feet physical distancing requirements (*8*). The study detected no teacher-driven transmission; other school investigations have identified teachers and staff members as being central to in-school transmission^¶¶¶¶^ (*9*,*10*). Although school-associated transmission was rare in this investigation, most cases did lead to household transmission, highlighting the importance of reducing school transmission to prevent infected children from transmitting SARS-CoV-2 to household members.

The modified quarantine policy, allowing contacts to continue attending in-person school if both the index patient and the contact were wearing a mask, did not lead to additional school-associated transmission and resulted in over 1,200 student in-person learning days saved.***** Among the five school-associated cases, the contact or index patient often had poor mask compliance, or they sat near one another during lunch. Findings suggest that quarantine determinations based on mask use of the index patient and close contacts might be adequate for preventing additional school-associated transmission in schools implementing multiple critical prevention strategies.

The findings in this report are subject to at least four limitations. First, WGS to differentiate school-associated from community transmission in a high incidence setting was not always available. Second, some infected contacts might have been missed because not all contacts received testing and the winter break mid-investigation might have interrupted additional school-associated transmission. Third, misclassification of susceptibility might have occurred as immunity status was unknown. Finally, these findings are specific to the current circulating SARS-CoV-2 variant distribution; as variant distribution shifts to new variants, more transmission might occur.

In an urban county with high SARS-CoV-2 community incidence, comprehensive testing of contacts detected low school-associated transmission in elementary schools, with a secondary attack rate of 0.7%. These results suggest that when ≥6 ft distancing is not feasible, schools in high-incidence communities can still limit in-school transmission by consistently using masks and implementing other important mitigation strategies.

**Summary**

**What is already known about this topic?**

Data suggest that school-associated SARS-CoV-2 transmission is low.

**What is added by this report?**

SARS-CoV-2 testing was offered to 1,041 school contacts of 51 index patients across 20 elementary schools in Salt Lake County, Utah. In a high community transmission setting, low school-associated transmission was observed with a 0.7% secondary attack rate. Mask adherence was high, but students’ classroom seats were <6 ft apart and a median of 3 ft apart.

**What are the implications for public health practice?**

These findings add to evidence that in-person elementary schools can be opened safely with minimal in-school transmission when critical prevention strategies including mask use are implemented, even though maintaining ≥6 ft between students’ seats might not be possible.

## Footnotes

* These authors contributed equally to this report.

† The 7-day cumulative number of new COVID-19 cases in Salt Lake County was obtained from the Utah Department of Health and the Salt Lake County Health Department.

§ Susceptible persons were defined as those with no record of previous positive test results for SARS-CoV-2 or whose date of laboratory-confirmed infection onset was at least 90 days earlier (https://www.cdc.gov/coronavirus/2019-ncov/hcp/duration-isolation.html).

¶ A school contact was defined as a student or staff member who was in contact with the index patient for a cumulative total of 15 minutes or more during a 24-hour period in a classroom, cafeteria, school bus, or recess space during an index patient’s infectious period.

** An index patient was defined as a student or staff member with laboratory-confirmed SARS-CoV-2 infection who had attended in-person school while infectious for at least 1 day. Infectious period was estimated as 2 days before to 10 days after date of symptom onset (if symptomatic) or date of first positive specimen collection (if asymptomatic) (https://www.cdc.gov/coronavirus/2019-ncov/php/contact-tracing/contact-tracing-plan/investigating-covid-19-case.html).

†† This school district consists of approximately 67,000 K–12 students and 7,500 employees at 63 elementary schools, 15 junior high schools, eight high schools, and other special schools. Once schools reopened, students were given the option to participate in a hybrid model (four days of in-person school and one day of online learning) or all online learning. Winter break occurred during December 21, 2020–January 1, 2021; in total, the investigation period encompassed 21 days of in-person learning.

§§ Students were placed in cohorts by classroom whenever possible to reduce interactions between classes. Most schools staggered lunch, gym classes, and special activities, such as library use or art classes. At some schools, classes would mix by grade level at recess. Schools limited nonessential extracurricular in-person events, and other events (e.g., sports, assemblies, performances, and field trips) were held virtually when feasible.

¶¶ The University of Utah’s HERO Project is sponsored by the Governor’s Office of Management and Budget and aims to provide data to aid in decision-making that allows a safe return to normal for Utah’s citizens and economy (https://eccles.utah.edu/utah-hero/).

*** SARS-CoV-2 genome sequences were assigned to global lineages with pangolin (v.2.1.10, pangoLEARN v.2021–02–01; https://github.com/cov-lineages/pangolin).

††† 45 C.F.R. part 46; 21 C.F.R. part 56; 42 U.S.C. Sect. 241(d), 5 U.S.C. Sect. 552a, 44 U.S.C. Sect. 3501 et seq.

§§§ An additional 52 school contacts had at least one household member with laboratory-confirmed SARS-CoV-2 during the preceding 90 days; these school contacts were still considered susceptible and eligible for inclusion, although they might have been previously infected and already immune.

¶¶¶ An outbreak was defined as two or more cases epidemiologically linked to the same index patient classroom.

**** The secondary attack rate excludes seven nonschool-associated cases from the numerator and the denominator. Among 105 quarantined school contacts who were tested, the secondary attack rate excludes four nonschool-associated cases. Among 630 non-quarantined school contacts who were tested, the secondary attack rate excludes three nonschool-associated cases.

†††† Persons could return to school without SARS-CoV-2 testing after a 10-day quarantine. Those who received a negative SARS-CoV-2 test result on quarantine days 7–9 could return to school early.

§§§§ Among the 51 index patients, 42 classroom teachers were surveyed. Six index patients did not have traditional classroom exposures and were excluded; five were teachers or staff members who circulated among multiple classrooms a day and interacted with students one-on-one or in small groups, and one was a student in a class for children with special health care needs. Three classrooms had two index patients; only one teacher’s survey was used to avoid double counting the classrooms.

¶¶¶¶ https://www.medrxiv.org/content/10.1101/2021.02.04.21250670v2.full***** This calculation assumes that each student would have missed 8 in-person school days because the students attended in-person learning four out of five school days a week. In addition, it also assumes that all 158 students who would have been quarantined in December but were not quarantined in January were not school-associated cases, although only 111 of 158 were tested for SARS-CoV-2.
