# *Erratum*: Vol. 70, No. 12

**DOI:** 10.15585/mmwr.mm7017a6

**Published:** 2021-04-30

In the report “Low SARS-CoV-2 Transmission in Elementary Schools — Salt Lake County, Utah, December 3, 2020–January 31, 2021,” on page 445, the fourth footnote of Table 2 should have read, “^¶ ^Restricted to students (n = 908).** The five students in grade 7 or higher were contacts of two index patients at the same school: three were exposed to an elementary school student on the school bus and two to a school staff member in a nonclassroom space at the school. **Bus contacts were not routinely included on the list of school contacts for all 51 index patients.”

